# Right hemisphere dominance directly predicts both baseline V1 cortical excitability and the degree of top-down modulation exerted over low-level brain structures

**DOI:** 10.1016/j.neuroscience.2015.10.045

**Published:** 2015-12-17

**Authors:** Q. Arshad, S. Siddiqui, S. Ramachandran, U. Goga, A. Bonsu, M. Patel, R.E. Roberts, Y. Nigmatullina, P. Malhotra, A.M. Bronstein

**Affiliations:** Division of Brain Sciences, Charing Cross Hospital Campus, Imperial College, Fulham Palace Road, London W6 8RF, UK

**Keywords:** tDCS, trans-cranial direct current stimulation, TMS, trans-cranial magnetic stimulation, VOR, vestibular-ocular reflex, right hemisphere dominance, visual cortical excitability, vestibular–ocular reflex, line bisection, top-down modulation

## Abstract

Right hemisphere dominance for visuo-spatial attention is characteristically observed in most right-handed individuals. This dominance has been attributed to both an anatomically larger right fronto-parietal network and the existence of asymmetric parietal interhemispheric connections. Previously it has been demonstrated that interhemispheric conflict, which induces left hemisphere inhibition, results in the modulation of both (i) the excitability of the early visual cortex (V1) and (ii) the brainstem-mediated vestibular–ocular reflex (VOR) via top-down control mechanisms. However to date, it remains unknown whether the degree of an individual’s right hemisphere dominance for visuospatial function can influence, (i) the baseline excitability of the visual cortex and (ii) the extent to which the right hemisphere can exert top-down modulation. We directly tested this by correlating line bisection error (or pseudoneglect), taken as a measure of right hemisphere dominance, with both (i) visual cortical excitability measured using phosphene perception elicited via single-pulse occipital trans-cranial magnetic stimulation (TMS) and (ii) the degree of trans-cranial direct current stimulation (tDCS)-mediated VOR suppression, following left hemisphere inhibition. We found that those individuals with greater right hemisphere dominance had a less excitable early visual cortex at baseline and demonstrated a greater degree of vestibular nystagmus suppression following left hemisphere cathodal tDCS. To conclude, our results provide the first demonstration that individual differences in right hemisphere dominance can directly predict both the baseline excitability of low-level brain structures and the degree of top-down modulation exerted over them.

## Highlights

• Line bisection predicts V1 excitability.
• Line bisection predicts degree of VOR modulation.
• Line bisection correlates with tDCS-mediated vestibular-nystagmus suppression.
• Degree of nystagmus suppression is a bio-marker of right hemisphere dominance.

## Introduction

Disrupting the right lateralized fronto-parietal attentional network to induce interhemispheric competition has previously been shown to modulate low-level brain function. Specifically, these modulatory effects have been shown to influence both visual cortex excitability and the brainstem-mediated vestibular–ocular reflex (VOR) (Silvanto et al., 2009, Arshad et al., 2013b) Such modulation was suggested to occur due to left hemisphere inhibition (Silvanto et al., 2009, Arshad et al., 2013b), owing in part to the fact that the right hemisphere is dominant for visuospatial function in right-handed individuals (Kinsbourne, 1977).

Right hemisphere dominance for spatial functions has been attributed to the existence of an anatomically larger right parieto-frontal network that correlates with visuo-spatial ability (De Schotten et al., 2011) and the presence of asymmetric parietal interhemispheric connections, which allows for the right hemisphere to exert a greater degree of inhibition (Koch et al., 2011). However to date, it remains unknown whether individual differences in the degree of right hemispheric dominance can influence (i) the baseline excitability of the visual cortex and (ii) the extent to which top-down modulation can be exerted over the brainstem-mediated VOR.

Herewith, we directly tested for this by correlating line bisection error (sometimes referred to as ‘pseudoneglect’), taken as a measure of right hemisphere dominance, with (i) baseline visual cortical excitability and (ii) the degree of vestibular nystagmus suppression following left hemisphere cathodal trans-cranial direct current stimulation (tDCS).

## Experimental procedures

### Subjects

Twenty-eight right-handed subjects (Handedness score over 40 (Oldfield, 1971) participated in the study (15 female, age range 19–24 years, mean age 22 years). Note, our power calculation revealed that in order to achieve a correlation coefficient of 0.65 and above, at an alpha value of 0.05 (two tails) and power of 0.9, would require 28 participants. All subjects were naive to the purpose of study and had no history of otological, ophthalmological, psychiatric or neurological disorders. All subjects provided written informed consent as approved by the local ethics research committee.

### Line bisection task

Each subject performed 10 line bisection trials. For each trial a single centralized 20-cm long and 1-mm wide black line was presented to the subject on a horizontally orientated A4 paper sheet. Subjects were required to bisect the line at the perceived mid-point with a red ballpoint pen. Five trials were performed with the right hand and the remaining five trials were performed with the left hand (randomized order). In each trial we calculated the deviation in mm from the true center (De Schotten et al., 2011). For each subject we summed the 10 individual deviation values and calculated the mean deviation. A positive line bisection error denoted a bias to the right of the true center, whereas a negative error denoted a bias to the left.

### Threshold measurement of visual cortical excitability

Application of single-pulse trans-cranial magnetic stimulation (TMS) over the occipital cortex elicits the illusionary percept of a brief flash of light. This illusionary percept is termed a phosphene and the intensity of TMS required to elicit these percept’s are suggested to reflect the underlying excitability of the visual cortex (Boroojerdi et al., 2002). That is, if low intensities of TMS (i.e. a low % of the total maximum stimulator output) can elicit phosphenes, it implies that the underlying cortical excitability is high and vice versa.

Biphasic TMS pulses were administrated with a Magstim 200 stimulator (Magstim Co, UK). We used a 70-mm figure of an eight-shaped coil, always held with the handle turned laterally. For V1 the coil was placed centrally over the inion until the brightest stationary phosphene was reported in the central visual field (Arshad et al., 2014a). For V5, TMS was applied 3 cm vertically up and 5 cm laterally (either right or left) from the inion until the brightest moving phosphene was detected (Seemungal et al., 2013). The participants were asked to verbally describe the phosphene and its perceived location in terms of its position superimposed upon an imagined clock face. Initially, the intensity of the stimulation was set at 68% of the maximum stimulator output, but this was increased if the participants did not perceive a phosphene.

Visual cortical thresholds were established by implementing a modified binary search (MOBS) paradigm. Adopting this paradigm allowed for us to determine the intensity of TMS required to elicit a phosphene 50% of the time (i.e., threshold). This paradigm utilizes an adaptive procedure, whereby the initial stimulus value (TMS pulse) is presented at a value which represents the bisection of an initial upper and lower boundary pair. These boundary pairs are continually updated based upon the subject’s prior response to each TMS pulse (e.g. a positive subjective response will shift the boundary downward and vice versa). The actual threshold was determined after subjects made three consecutive alternate choices in order to minimize variability. Once, we had established the threshold we ascertained whether it was correct by applying a trial sequence of 20 TMS pulses. Each of the 20 TMS pulses were separated by 6 s and the subject had to respond verbally with either a “yes” or “no” response to whether they had perceived a phosphene following each TMS pulse. If the established threshold was correct then we observed 10 (+ or − 2 i.e. between 8 and 12) yes responses (Arshad et al., 2014a). If the number of “yes” responses was outside this range, the threshold was re-established.

### Vestibular stimulation and eye movement recording

Following otoscopy to exclude local contra-indications, subjects underwent caloric stimulation to elicit the vestibular-ocular reflex (VOR). Participants lay supine on a couch with the head tilted up by 30° in order to obtain maximal horizontal semi-circular canal activation. Caloric irrigations were performed with cold water (i.e. 30 °C; 7 °C below core body temperature) with a flow rate of 500 ml/min for 40 s (CHARTR VNG; ICS medical). In response to the caloric stimulation an oculomotor response is elicited in the form of ‘vestibular nystagmus’. The onset of nystagmus and the perceptual state of vertigo typically begins approximately 20 s after the start of the irrigation reaching a peak at around 60 s. The total duration of the response lasts on average 3 min in total (Fitzgerald and Hallpike, 1942).

Right cold caloric irrigations induce left-beating vestibular nystagmus (i.e. beating toward the non-stimulated ear) with a rightward slow phase component. Conversely, left cold caloric irrigations elicit a right-beating vestibular nystagmus. Eye movements were recorded using a head-mounted infra-red binocular video-oculography (VOG) system (CHARTR VNG; ICS medical). Eye movements were analyzed using a computerized automatic analysis program (CHARTR VNG; ICS medical) that removed the fast phases of the nystagmus and plotted each individual slow phase velocity over a period of 100 s. The response intensity was determined by identifying the peak slow phase eye velocity.

### tDCS

Stimulation was applied using a battery-driven stimulator (neuroConn GMBH, ilmenau, Germany). We applied either unipolar cathodal (test condition) or anodal (control condition) tDCS, over the left hemisphere (P3: international 10–20 system for EEG electrode placement; electrode placement area 25 cm^2^). The reference electrode was always placed over the ipsilateral shoulder, with a larger electrode placement area of 35cm^2^ in order to maximize subject comfort. This specific stimulation montage was employed as our previous work has shown that left hemisphere cathodal stimulation induces vestibular nystagmus suppression. Left hemisphere anodal stimulation has previously been shown to have no modulatory effect upon the VOR; thereby implementing this condition allowed for us to control for non-specific effects that could be attributed to electrical stimulation. The current had a ramp-up time of 10 s at which point a constant current with an intensity of 1.5 mA was applied for a total duration of 15 min. The current ramped down in a 10-s fade-out period (Arshad et al., 2014b, Arshad et al., 2015).

**Fig. 1:**
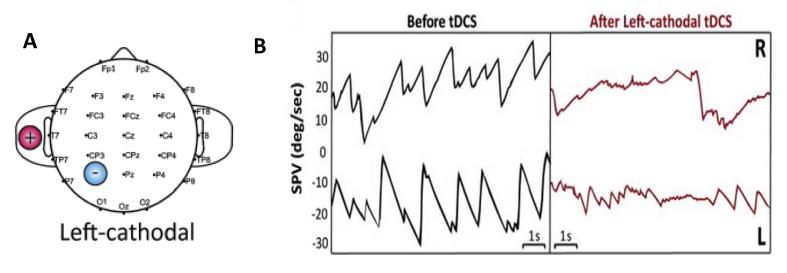
(A) tDCS montage implemented to induce vestibular suppression, as previously described in Arshad et al. (2014b). Unipolar left cathodal stimulation was applied for a duration of 15 min with a current of 1.5 Ma. The cathode electrode was applied over left P3 (international 10–20 system for EEG electrode placement); electrode placement area 25 cm^2^. The reference electrode was always placed on the ipsilateral shoulder. (B) Illustrates the effects of left cathodal tDCS upon vestibular nystagmus. In the left panel (black trace) we show the raw trace depicting the vestibular nystagmus elicited during right (upper trace) and left (lower trace) ear cold water caloric irrigations. Right ear cold irrigations induce a left-beating vestibular nystagmus (downward deflection of the fast phase) whereas left ear cold irrigations induce a right-beating vestibular nystagmus (upward deflection of the fast phase. Note the marked suppression of vestibular nystagmus as shown in the right panel (red trace; shown for an individual subject) following left hemisphere inhibition for both right and left ear cold water irrigations. (For interpretation of the references to color in this figure legend, the reader is referred to the web version of this article.)

Cold water caloric irrigations (one per ear; right and left ear in randomized order) were performed to establish the peak SPV before and after either cathodal or anodal tDCS over the left posterior parietal cortex. We calculated the mean % change in peak slow phase velocity before and after tDCS. It is important to note that we only expected to observe a change in the VOR following left cathodal tDCS as shown in Fig. 1, and importantly not following left anodal stimulation which we included as a control condition.

### Experimental protocol

For each subject we initially performed the 10-line bisection trials to establish the degree of baseline hemispheric asymmetry at rest in a well-lit office. Subsequently, in a darkened room we established V1 and V5 (both right and left; randomized order between cortical loci) visual cortical thresholds using TMS as described above.

After a one-hour rest interval to avoid any potential TMS-related carry over effects, subjects underwent 2 cold water caloric irrigations (i.e. right and left ear (randomized order). Note, in accordance with the American National Standards Institute guidelines, each irrigation was separated by 5 min from the end of the eye movement recording (i.e. 3 min post-irrigation). These initial two irrigations allowed for us to establish the pre tDCS peak slow phase eye velocity (SPV). Following this, either left hemisphere cathodal or anodal tDCS was applied for a total duration of 15 min as detailed above. After tDCS the caloric irrigations were repeated to establish the post tDCS peak SPV.

## Results

(i) Relationship between line bisection error and visual cortical thresholds

Mean visual cortical thresholds represented as percentages of the maximum TMS stimulator output were 49.8% SD = 12.0 (range 28–72%) for V1, 53.5% SD = 10.14 (range 34–72%) for right V5 and 54.8% SD = 8.69 (range 40–74%) for left V5. Mean line bisection error was −2.62 mm SD = 2.89.

**Fig. 2:**
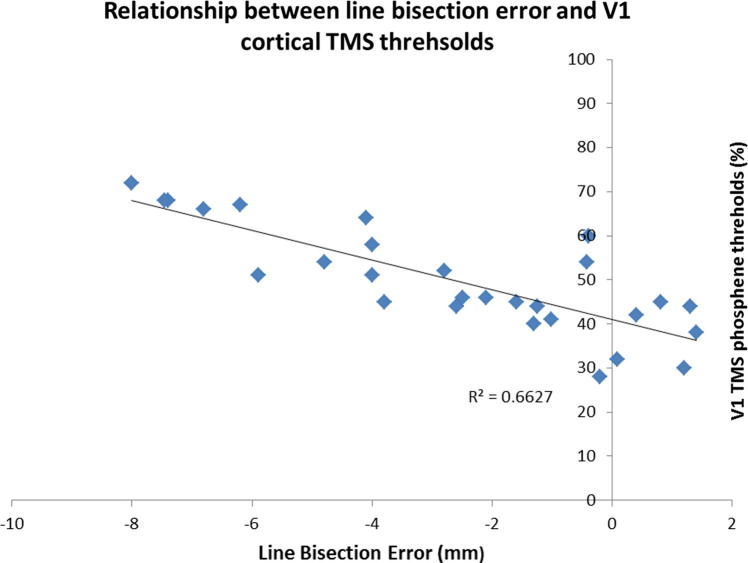
Significant negative correlation between the line bisection error (mm) and V1 cortical thresholds as measured by TMS. Subjects with lower visual cortical TMS thresholds (i.e. more excitable visual cortex) demonstrated greater shifts during the line bisection task to the right of the true center (i.e. positive line bisection error). In contrast, those individuals that were more right hemisphere dominant as reflected by a more negative line bisection error had higher visual cortical thresholds (i.e. lower cortical excitability).

A significant negative correlation was observed between line bisection error and V1 cortical thresholds (*R*^2^ = 0.6627, *p* < 0.01, Pearson’s correlation) (Fig. 2). No significant correlations were observed for either right or left V5 (*R*^2^ = 0.023, *p* > 0.05 and *R*^2^ = 0.016, *p* > 0.05) respectively.

(ii) Relationship between line bisection error and tDCS-mediated vestibular nystagmus suppression

**Fig. 3:**
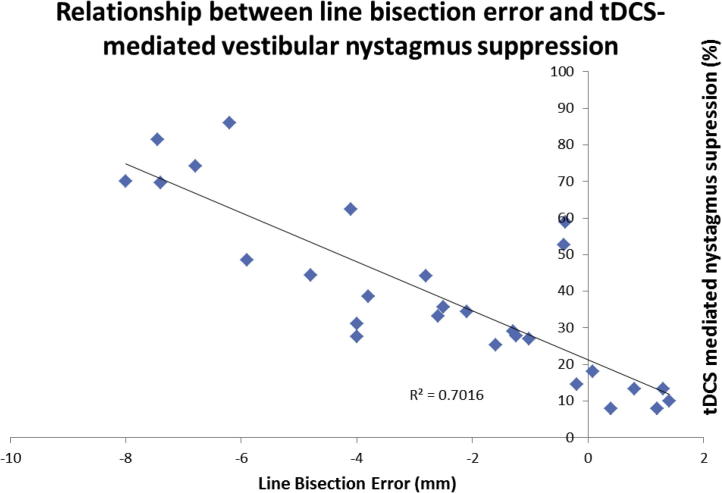
Significant negative correlation between line bisection error (mm) and mean % change in peak SPV of caloric induced nystagmus following left cathodal tDCS. Those subjects that were more right hemisphere dominant (i.e. more negative line bisection error) demonstrated larger mean % changes in the VOR following left hemisphere inhibition.

The mean % change in the VOR (i.e. vestibular nystagmus suppression) following left cathodal tDCS was −38.8% (range −7.8% to −86.0%). A significant negative correlation was observed between line bisection error and the mean % change in SPV following left cathodal tDCS (*R*^2^ = 0.7016, *p* < 0.01 Pearson’s correlation) (Fig. 3). Following left anodal tDCS (control condition) the mean % change in the VOR was + 2.2% (range +5.8% to −8.2%). No correlation was observed between line bisection error and the mean % change in SPV following left anodal tDCS (*R*^2^ = 0.014, *p* > 0.05 Pearson’s correlation).

(iii) Relationship between tDCS-mediated vestibular nystagmus suppression and visual cortical thresholds

**Fig. 4:**
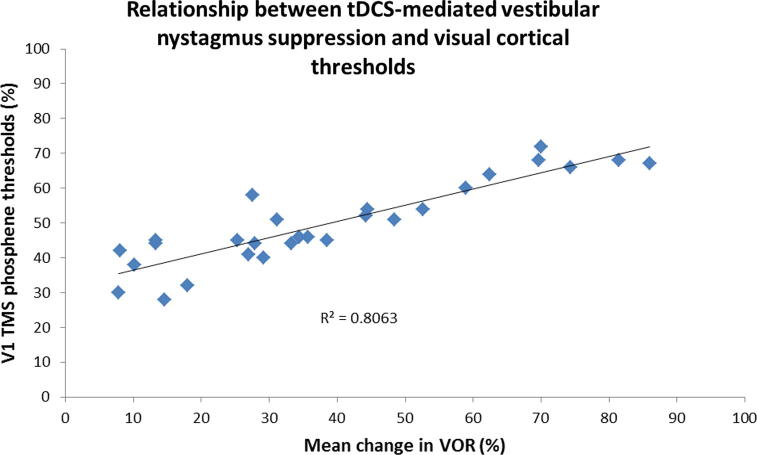
Significant correlation between mean % change in the VOR following left hemisphere inhibition with V1 cortical thresholds. Individuals who were more right hemisphere dominant, i.e. larger mean % change in the VOR, had higher thresholds (i.e. less excitable visual cortex) in comparison to those individuals who were less right hemisphere dominant (i.e. lower thresholds).

To ascertain whether line bisection error could be substituted with tDCS-mediated vestibular nystagmus suppression as an objective physiological bio-marker of right hemisphere dominance we correlated the mean % change in SPV following left cathodal tDCS with visual cortical thresholds. We observed a significant positive correlation between the mean % change in SPV with V1 cortical thresholds *R*^2^ = 0.8063, *p* < 0.01 (Fig. 4).No correlation was observed for either right or left V5 (*R*^2^ = 0.123, *p* > 0.05 and *R*^2^ = 0.074, *p* > 0.05) respectively.

## Discussion

We found that those individuals with greater right hemisphere dominance had a less excitable early visual cortex at the baseline and were able to exert more top-down modulation over the brainstem-mediated VOR.

Firstly, we consider why those individuals with greater right hemisphere dominance might have a less excitable early visual cortex? Previous work has demonstrated that the early visual cortex is particularly susceptible to top-down modulation (Watanabe et al., 2011). Specifically, in normal subjects it has been shown that following interhemispheric competition in the attentional network, induced via concurrent vestibular simulation and the performance of a visuo-spatial working memory task, results in decreased visual cortical excitability (Arshad et al., 2013b). In contrast, Silvanto and colleagues demonstrated that inducing interhemispheric competition within attention networks with focal TMS resulted in increased V1 excitability (Silvanto et al., 2009), implying excitatory rather than inhibitory top-down modulatory effects upon V1. We suggest that the contrasting results are due to methodological differences of inducing interhemispheric competition. That is, TMS is a highly focal technique capable of targeting specific anatomical loci within the PPC (Silvanto et al., 2009), whereas the behavioral manipulation we previously employed (i.e. vestibular stimulation combined with a visuo-spatial task) is likely to affect the fronto-parietal attention network in a more global manner. In any case, modifying activity within the right-lateralized attention network through increased interhemispheric competition appears to modulate V1 excitability via top-down control (Silvanto et al., 2009, Arshad et al., 2013b). Our current findings extend this previous knowledge by demonstrating that at rest, baseline excitability of V1 is directly related to the extent of an individual’s right hemisphere dominance, presumably mediated by inhibitory projections (Arshad et al., 2013b). That is, those individuals with greater right hemisphere dominance for visuo-spatial function, as reflected by a more negative line bisection error, have a less excitable visual cortex (i.e. elevated thresholds), possibly attributable to the existence of stronger inhibitory cortical projections in those individuals.

We shall now turn to consider the relationship found between line bisection error and the degree of tDCS-mediated vestibular nystagmus suppression. We found that those individuals who were more right hemisphere dominant suppressed vestibular nystagmus more strongly. As left cathodal tDCS inhibits the left hemisphere (Arshad et al., 2014b), it is proposed that as a result the right hemisphere becomes hyperactive (Sparing et al., 2009). Accordingly, those individuals who are more right hemisphere dominant, exhibit comparatively greater hyperactivation, and consequently inhibit the left hemisphere more strongly, in turn inducing greater VOR suppression (Szczepanski et al., 2010, Szczepanski and Kastner, 2013). Previously we have shown that, in right-handers, right hemisphere inhibition induces no modulatory effect upon the VOR (Arshad et al., 2015). The absence of a modulatory effect upon the VOR was postulated to be attributable to the right hemisphere’s dominance for vestibular processing (Dieterich et al., 2003, Arshad et al., 2013a). An alternative, but not a mutually exclusive, explanation is that it may be attributable to the right hemisphere’s dominance for spatial processing (Kinsbourne, 1977) mediated by the on-going functional asymmetries between the two parietal cortices, which are facilitated by asymmetric parietal interhemispheric connections (Koch et al., 2011). In further support of this view, we have recently found that in left-handed individuals, right- but not left hemisphere cathodal tDCS modulates the VOR, as these individuals have left hemisphere dominance for vestibular processing, (Arshad et al., 2015).

Finally, we assessed whether tDCS-mediated VOR suppression could potentially be implemented as an objective physiological biomarker of right hemisphere dominance. We found that tDCS-mediated VOR suppression correlated with the baseline excitability of the early (V1) but not late (V5) visual cortex, similar to that found when we correlated line bisection error with visual cortical thresholds. This finding implies that tDCS-mediated VOR suppression is a potential biomarker of right hemisphere dominance. Further, such a measure would not be as susceptible to environmental and stimulus factors in the same way as line bisection (Jewell and McCourt, 2000).

## Conclusion

We provide a novel demonstration that more right hemisphere dominant individuals have a less excitable early visual cortex and exert stronger top-down influences over the brainstem-mediated VOR. Additionally, we demonstrate that tDCS-mediated VOR suppression following left hemisphere inhibition could be implemented as a novel index for interhemispheric asymmetry and hemispheric dominance. Although, we have only explored its relationship to asymmetries in healthy individuals, it will be critical in the future to examine whether it can also reflect the pathological asymmetries that are caused by unilateral brain damage. Crucially, if this is the case it has the potential to be employed as a biomarker of recovery and a measure of plasticity in clinical populations.

## Conflict of interest

The authors report no conflict of interest.
